# Circular RNA ITCH promotes extracellular matrix degradation via activating Wnt/β-catenin signaling in intervertebral disc degeneration

**DOI:** 10.18632/aging.203036

**Published:** 2021-05-18

**Authors:** Feng Zhang, Feili Lin, Zhiwen Xu, Zheng Huang

**Affiliations:** 1 Department of Orthopaedic, The Second Affiliated Hospital of Zhejiang University School of Medicine, Hangzhou, Zhejiang Province, China; 2 Department of Nephrology, The Second Affiliated Hospital of Zhejiang University School of Medicine, Hangzhou, Zhejiang Province, China; 3 Department of Orthopaedic, The Fourth Affiliated Hospital of Zhejiang University School of Medicine, Hangzhou, Zhejiang Province, China; 4 Guanghua Hospital Affiliated to Shanghai University of Traditional Chinese Medicine, Shanghai, China

**Keywords:** intervertebral disc degeneration, extracellular matrix ECM degradation, circITCH, miR-17-5p, SOX4

## Abstract

Intervertebral disc degeneration (IDD) is the prevailing spine disorder and is associated with musculoskeletal disease. The extracellular matrix (ECM) degradation is an essential hallmark of IDD progression. Circular RNAs (circRNAs), as crucial cellular regulators, participate in multiple pathological processes including IDD. Here, we tried to explore the effect of circITCH on the ECM degradation of IDD and the underlying mechanism. Significantly, the expression levels of circITCH were elevated in the IDD patients’ nucleus pulposus (NP) tissues relative to that of normal cases. CircITCH promoted apoptosis and decreased proliferation of NP cells. CircITCH contributed to ECM degradation, as demonstrated by increased ADAMTS4 and MMP13 expression and decreased aggrecan and collagen II expression. Mechanically, miR-17-5p could be sponged by circITCH and miR-17-5p inhibited ECM degradation by repressing SOX4 in degenerative NP cells. CircITCH could activate Wnt/β-catenin pathway by targeting miR-17-5p/SOX4 signaling. SOX4 overexpression, miR-17-5p inhibitor, or Wnt/β-catenin signaling activator LiCl was able to reverse circITCH knockdown-inhibited apoptosis and ECM degradation, and circITCH knockdown-enhanced proliferation in NP cells. Thus, we conclude that circITCH promotes ECM degradation in IDD by activating Wnt/β-catenin through miR-17-5p/SOX4 signaling. Our finding presents novel insight into the mechanism that circITCH modulates the IDD progression. CircITCH and SOX4 may serve as potential targets for IDD therapy.

## INTRODUCTION

Low back pain is a common disease and approximately 80% of people experience this disease in their lifetime, leading to social and health burden [1]. The current etiology connects back pain with intervertebral disc degeneration (IDD) [2, 3]. The intervertebral disc (IVD) comprises the internal nucleus pulposus and peripheral annulus fibrosus [4, 5]. IDD generally presents extracellular matrix (ECM) reconstruction and a large amount of apoptosis [6]. Therefore, the understanding of the modulation mechanism of ECM degeneration is critical for developing practical therapeutic strategy of IDD and is urgently needed.

Circular RNAs (circRNAs) serve as an emerging kind of regulatory RNAs that forms a loop structure without 5′-3′ polyadenylated or polarities tails [7]. Most of the circRNAs show a more considerable stability degree than linear RNAs and are conserved among different species [8]. CircRNAs primarily derive from exons by a back-splice process. CircRNAs have been identified to interact with miRNA by functioning as competitive endogenous RNAs (ceRNAs) of miRNAs and affect the target mRNA expression [4, 9]. Meanwhile, some investigations have reported several circRNAs in modulating IDD. For example, CircFAM169A modulates IDD by regulating the miR-583/BTRC axis [10]. CircGRB10 is involved in the molecular circuits repressing human IDD [11]. Moreover, the role of circular RNA ITCH (circITCH) in cancer progression has been well identified. circITCH is able to induce cell apoptosis and inhibits cell proliferation in multiple cancer models [12, 13]. However, the role of circITCH in IDD regulation remains unreported.

MicroRNAs (miRNAs) are 17-25 nucleotides small-non-coding RNA and present important functions in different biological processes, including differentiation, proliferation, and apoptosis, by regulating various gene expressions [14, 15]. Meanwhile, miRNAs play important functions in the modulation of IDD development. It has been found that the miR-154 elevation enhances the ECM degradation of IDD [16]. CircSEMA4B regulated miR-431 modulating IL-1β-related degradative variations in IDD by Wnt signaling [17]. MiR-221 attenuates the protective influence of estrogen on IDD by targeting ER-α [18]. MiR-222-3p increases the development of IDD by regulating CDKN1B [19]. MiR-2355-5p represses ERFFI1 in restraining inflammation and proliferation of NP cells [20]. Besides, the function of miR-17-5p has been reported in multiple diseases, such as spinal cord injury, hypoxic-ischemic brain injury, and cancer [21–23]. Moreover, Wnt/β-catenin activation in IDD progression can enhance ECM [24]. Besides, several studies have well-recognized that the inhibition of sex-determining region Y-related high mobility group box 4 (SOX4) represses the Wnt/β-catenin pathway [25]. However, the correlation of circITCH with miR-17-5p and SOX4 in the modulation of ECM of IDD development remains elusive.

Here, we were interested in the exploration of circITCH functions in the modulation of IDD. We discovered a novel function of circITCH in promoting the ECM degradation of IDD by targeting miR-17-5p/SOX4/Wnt/β-catenin axis.

## RESULTS

### CircITCH induces NP cell apoptosis and reduces NP cell proliferation

**Figure 1:**
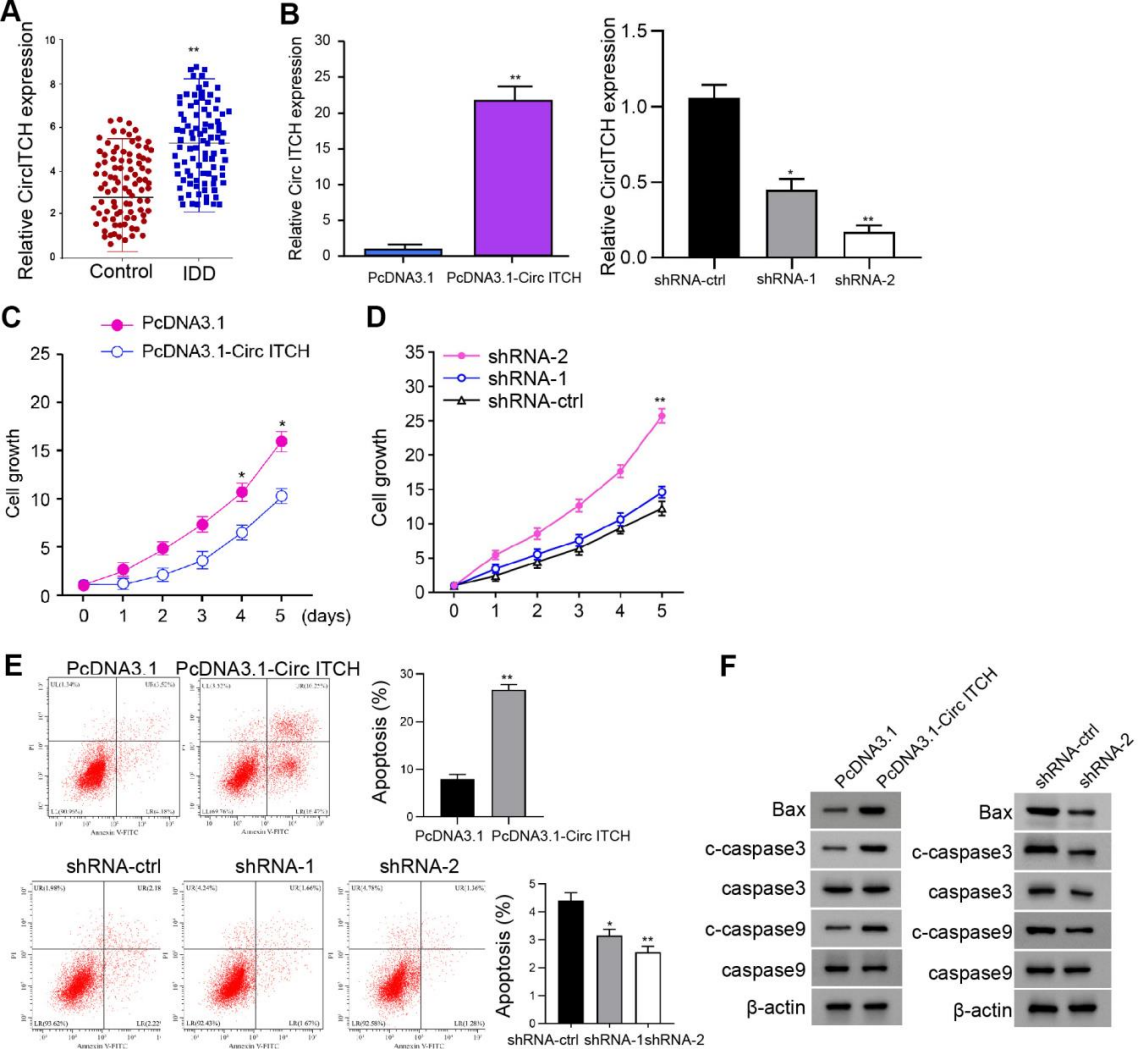
**CircITCH inhibits the proliferation and induces apoptosis of NP cells.** (**A**) The expression levels of circITCH were measured by qPCR in the NP tissues of IDD patients (n=90) and normal cases (n=90). (**B**–**F**) The NP cells were infected with lentiviral plasmids carrying circITCH shRNA or corresponding control shRNA or transfected with the pcDNA3.1 or the pcDNA3.1-circITCH overexpression vector. (**B**) The expression levels of circITCH were examined by qPCR in the cells. (**C**, **D**) The cell proliferation was analyzed by CCK-8 assays in the cells. (**E**) Cell apoptosis was tested by flow cytometry analysis in the cells. (**F**) The expression of Bax, caspase3, cleaved caspase3 (c-caspase3), caspase9, and cleaved caspase9 (c-caspase9) was measured by Western blot analysis. Data are presented as mean ± SD. Statistic significant differences were indicated: * *P* < 0.05.

For the evaluation of the potential association of circITCH with IDD, we analyzed the expression of circITCH in IDD patients and related normal subjects. CircITCH expression was elevated in NP tissue of IDD patients (n=90) relative to that of normal cases (n=90) (Figure 1A). Given that abnormal circITCH expression in the nucleus pulposus tissues of IDD patients, we analyzed the impact of circITCH on NP cell apoptosis and proliferation. To this end, the NP cells were infected with lentiviral plasmids carrying circITCH shRNA or corresponding control shRNA or transfected with the control vector or the circITCH overexpression vector. The efficiency of circITCH depletion and overexpression was validated in the cells (Figure 1B). CCK-8 assays showed that the NP cell proliferation was inhibited by circITCH overexpression but enhanced by circITCH knockdown (Figure 1C, 1D), indicating that circITCH inhibits NP cell proliferation. Moreover, the overexpression of circITCH enhanced while circITCH knockdown reduced apoptosis of NP cells (Figure 1E), suggesting that circITCH is able to induce NP cell apoptosis. We found that circITCH shRNA-2 presented a higher efficiency, which was selected in the subsequent experiments. Moreover, circITCH overexpression increased while circITCH knockdown decreased Bax, cleaved caspase3, and cleaved caspase9 expression in NP cells (Figure 1F).

### CircITCH promotes ECM degradation of degenerative NP cells

**Figure 2:**
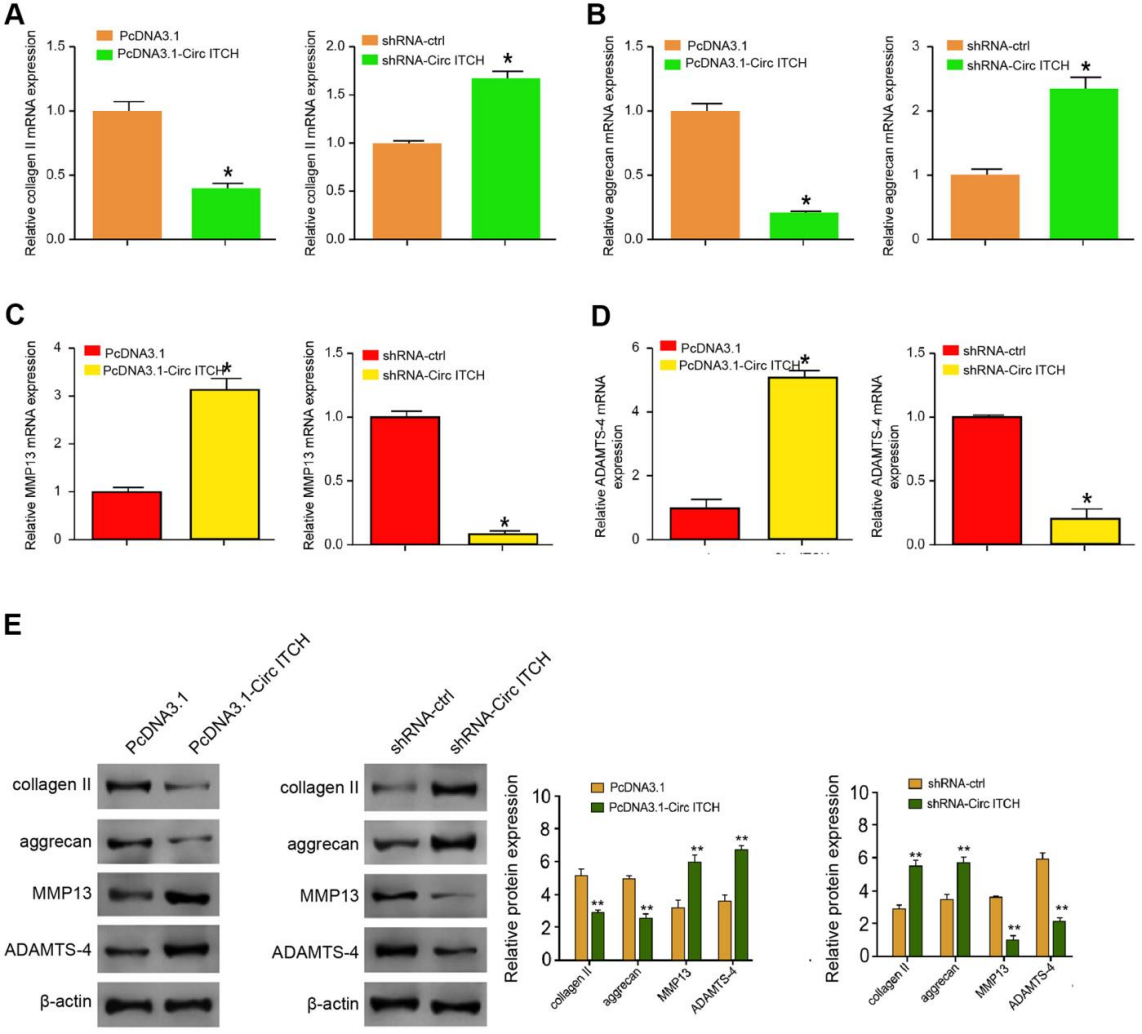
**CircITCH promotes ECM degradation of degenerative NP cells.** (**A**–**E**) The NP cells were infected with lentiviral plasmids carrying circITCH shRNA or corresponding control shRNA or transfected with the pcDNA3.1 or the pcDNA3.1-circITCH overexpression vector. (**A**–**D**) The mRNA expression of collagen II (**A**), aggrecan (**B**), MMP13 (**C**), and ADAMTS4 (**D**) was measured by qPCR in the cells. (**E**) The protein expression of collagen II, aggrecan, MMP13, ADAMTS4, and β-actin was tested by Western blot analysis in the cells. Data are presented as mean ± SD. Statistic significant differences were indicated: * *P* < 0.05.

To assess the role of circITCH in the modulation of extracellular matrix (ECM) degradation, the NP cells were infected with lentiviral plasmids carrying circITCH shRNA or corresponding control shRNA or transfected with the control vector or the circITCH overexpression vector. We analyzed the cellular expression of EMC degradation biomarkers, including aggrecan, collagen II, MMP13, and ADAMTS4. Significantly, the levels of aggrecan and collagen II were inhibited by circITCH overexpression but enhanced by the circITCH knockdown in the cells (Figure 2A, 2B). The overexpression of circITCH increased while the circITCH knockdown reduced the expression of MMP13 and ADAMTS4 in the cells (Figure 2C, 2D). Similarly, Western blot analysis presented the similar results (Figure 2E). Together these data suggest that circITCH promotes ECM degradation in NP cells.

### CircITCH is able to sponge miR-17-5p in NP cells

**Figure 3:**
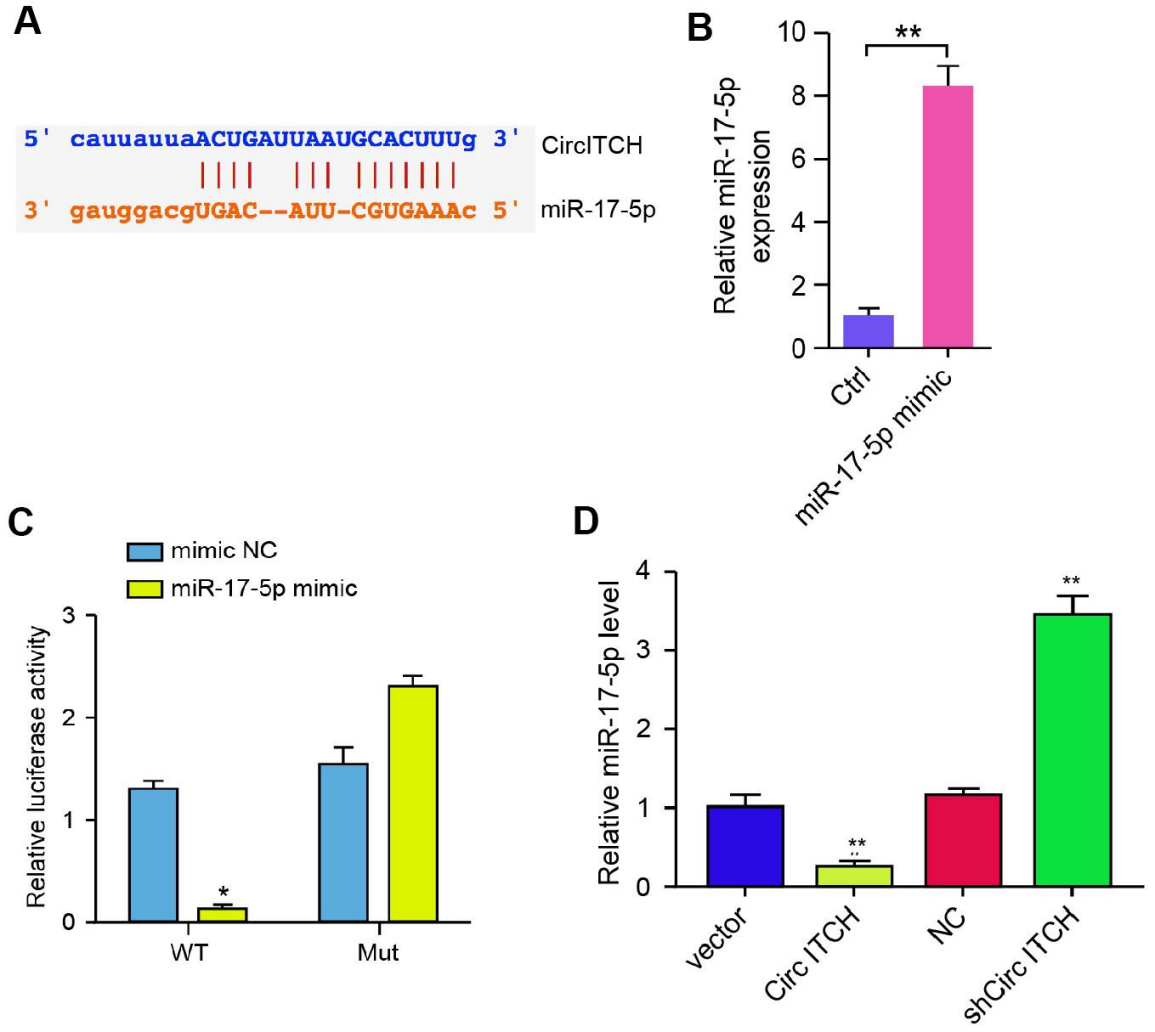
**CircITCH serves as a miR-17-5p sponge in NP cells.** (**A**) The potential interaction between circITCH and miR-17-5p was identified by the bioinformatic analysis using ENCORI (http://starbase.sysu.edu.cn/index.php). (**B**, **C**) The NP cells were treated with the miR-17-5p mimic or control mimic. (**B**) The expression levels of miR-17-5p were measured by qPCR in the cells. (**C**) The luciferase activities of wild type circITCH (circITCH WT) and circITCH with the miR-17-5p-binding site mutant (circITCH MUT) were determined by luciferase reporter gene assays in the cells. (**D**) The NP cells were infected with lentiviral plasmids carrying circITCH shRNA or corresponding control shRNA or transfected with the pcDNA3.1 or the pcDNA3.1-circITCH overexpression vector. The expression of miR-17-5p was analyzed by qPCR in the cells. Data are presented as mean ± SD. Statistic significant differences were indicated: * *P* < 0.05, ** *P* < 0.01.

Next, we explored the mechanism of circITCH-mediated ECM degradation in NP cells. We identified the potential interaction between circITCH and miR-17-5p in the bioinformatic analysis (Figure 3A). Then, we treated the NP cells with miR-17-5p mimic, and the efficiency was verified in the cells (Figure 3B). Remarkably, the miR-17-5p mimic reduced the luciferase activities of circITCH but not the circITCH mutant (Figure 3C). Moreover, NP cells were infected with lentiviral plasmids carrying circITCH shRNA or corresponding control shRNA or transfected with circITCH overexpression vector. Notably, the overexpression of circITCH reduced, but the depletion of circITCH enhanced the expression of miR-17-5p in the cells (Figure 3D). Together these data suggest that circITCH is able to sponge miR-17-5p in NP cells.

### MiR-17-5p inhibits ECM degradation in the degenerative NP cells

**Figure 4:**
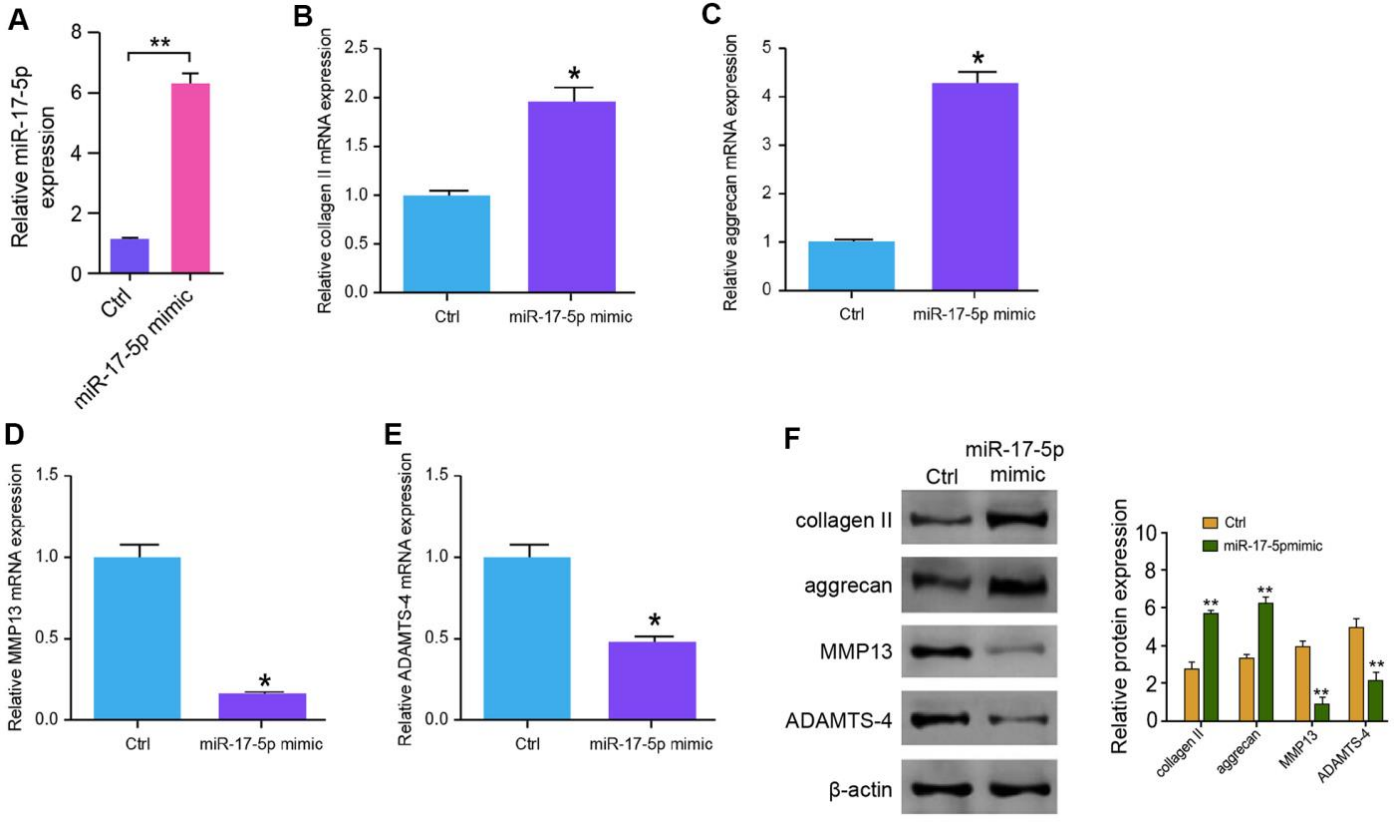
**MiR-17-5p inhibits ECM degradation in the degenerative NP cells.** (**A**–**F**) The NP cells were treated with the miR-17-5p mimic or control mimic. (**A**) The expression levels of miR-17-5p were tested by qPCR in the cells. (**B**–**E**) The mRNA expression of collagen II (**B**), aggrecan (**C**), MMP13 (**D**), and ADAMTS4 (**E**) was measured by qPCR in the cells. (**F**) The protein expression of collagen II, aggrecan, MMP13, ADAMTS4, and β-actin was determined by Western blot analysis in the cells. Data are presented as mean ± SD. Statistic significant differences were indicated: * *P* < 0.05, ** *P* < 0.01.

Then, we investigated further the functions of miR-17-5p in modulation of ECM degradation. NP cells were transfected with miR-17-5p mimic, and the depletion was validated in the cells (Figure 4A). The expression levels of collagen II and aggrecan were up-regulated by miR-17-5p in NP cells (Figure 4B, 4C). The treatment of miR-17-5p mimic notably inhibited the expression of MMP13 and ADAMTS4 in the cells (Figure 4D, 4E). Similarly, Western blot analysis demonstrated the similar results in the system (Figure 4F), suggesting that miR-17-5p inhibits ECM degradation in the degenerative NP cells.

### MiR-17-5p targets SOX4 in NP cells

**Figure 5:**
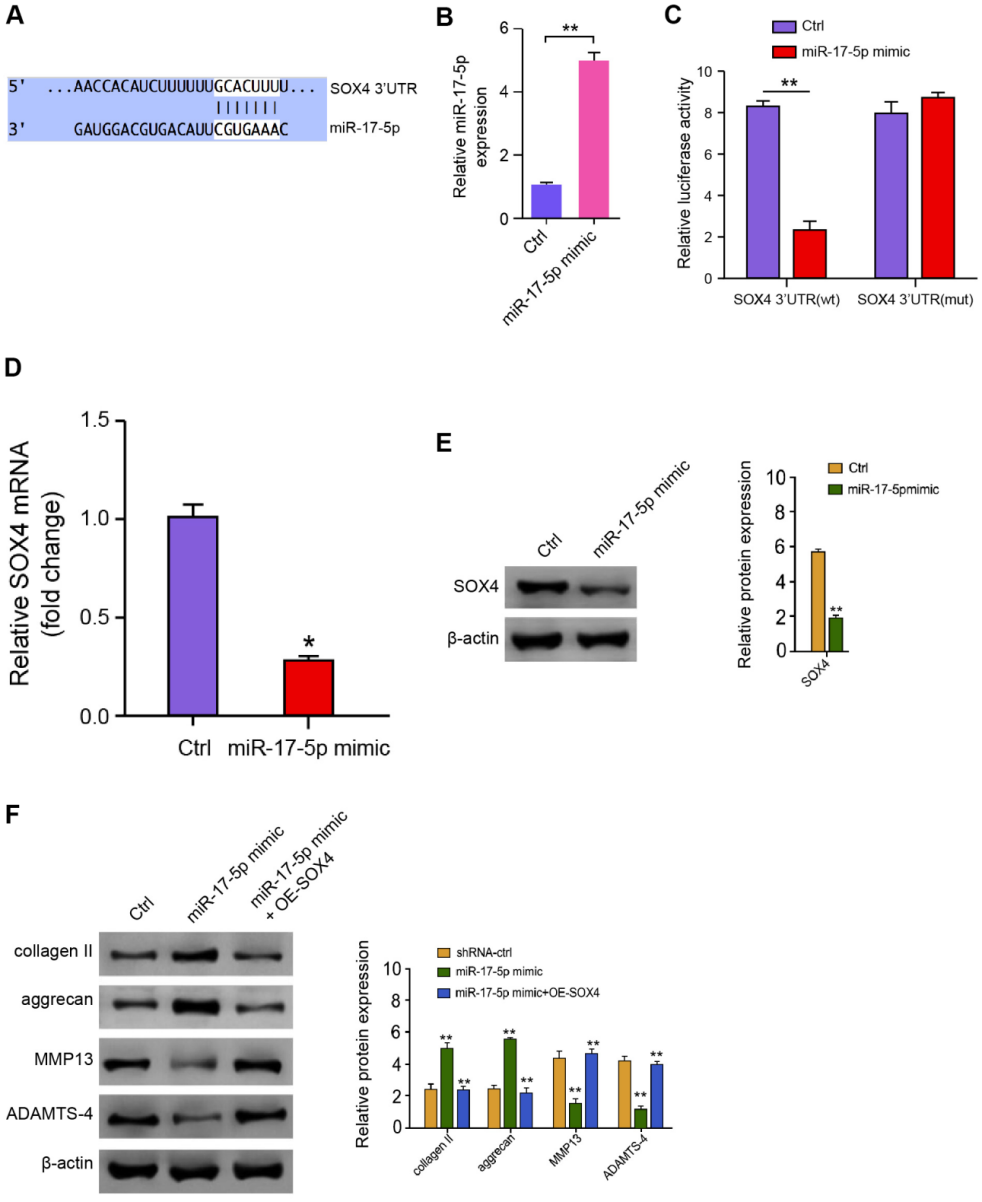
**MiR-17-5p targets SOX4 in NP cells.** (**A**) The interaction of miR-17-5p and SOX4 3’ UTR was identified by bioinformatic analysis using Targetscan (http://www.targetscan.org/vert_72/). (**B**–**E**) The NP cells were treated with the miR-17-5p mimic or control mimic. (**B**) The expression levels of miR-17-5p were measured by qPCR in the cells. (**C**) The luciferase activities of wild type SOX4 (SOX4 WT) and SOX4 with the miR-17-5p-binding site mutant (SOX4 MUT) were determined by luciferase reporter gene assays in the cell. (**D**) The mRNA expression of SOX4 was analyzed by qPCR in the cells. (**E**) The protein expression of SOX4 and β-actin was tested by Western blot analysis in the cells. (**F**) The protein expression of collagen II, aggrecan, MMP13, ADAMTS4, and β-actin was analyzed by Western blot analysis in the NP cells treated with control mimic, miR-17-5p mimic, or co-treated with miR-17-5p mimic and pcDNA3.1-SOX4 overexpression vector. Data are presented as mean ± SD. Statistic significant differences were indicated: * *P* < 0.05, ** *P* < 0.01.

We then discovered the mechanism of miR-17-5p-regulated ECM degradation in the degenerative NP cells. We identified the miR-17-5p-targeted site in SOX4 3’ UTR based on bioinformatic analysis (Figure 5A). To determine the influence of miR-17-5p on SOX4, we treated the NP cells with miR-17-5p mimic, and the efficiency was validated in the cells (Figure 5B). Significantly, the miR-17-5p mimic treatment attenuated luciferase activities of wild type SOX4 but not the SOX4 mutant in the NP cells (Figure 5C). Furthermore, the mRNA and protein expression of SOX4 were significantly repressed by miR-17-5p mimic in the NP cells (Figure 5D, 5E), suggesting that miR-17-5p is able to target SOX4 in the NP cells. Moreover, miR-17-5p mimic elevated the expression of collagen II and aggrecan while inhibited the expression of MMP13 and ADAMTS4, in which the overexpression of SOX4 could reverse these effects in the NP cells (Figure 5F), implying that miR-17-5p attenuates ECM degradation by targeting SOX4 in the NP cells.

### CircITCH activates Wnt/β-catenin pathway by targeting miR-17-5p/SOX4

**Figure 6:**
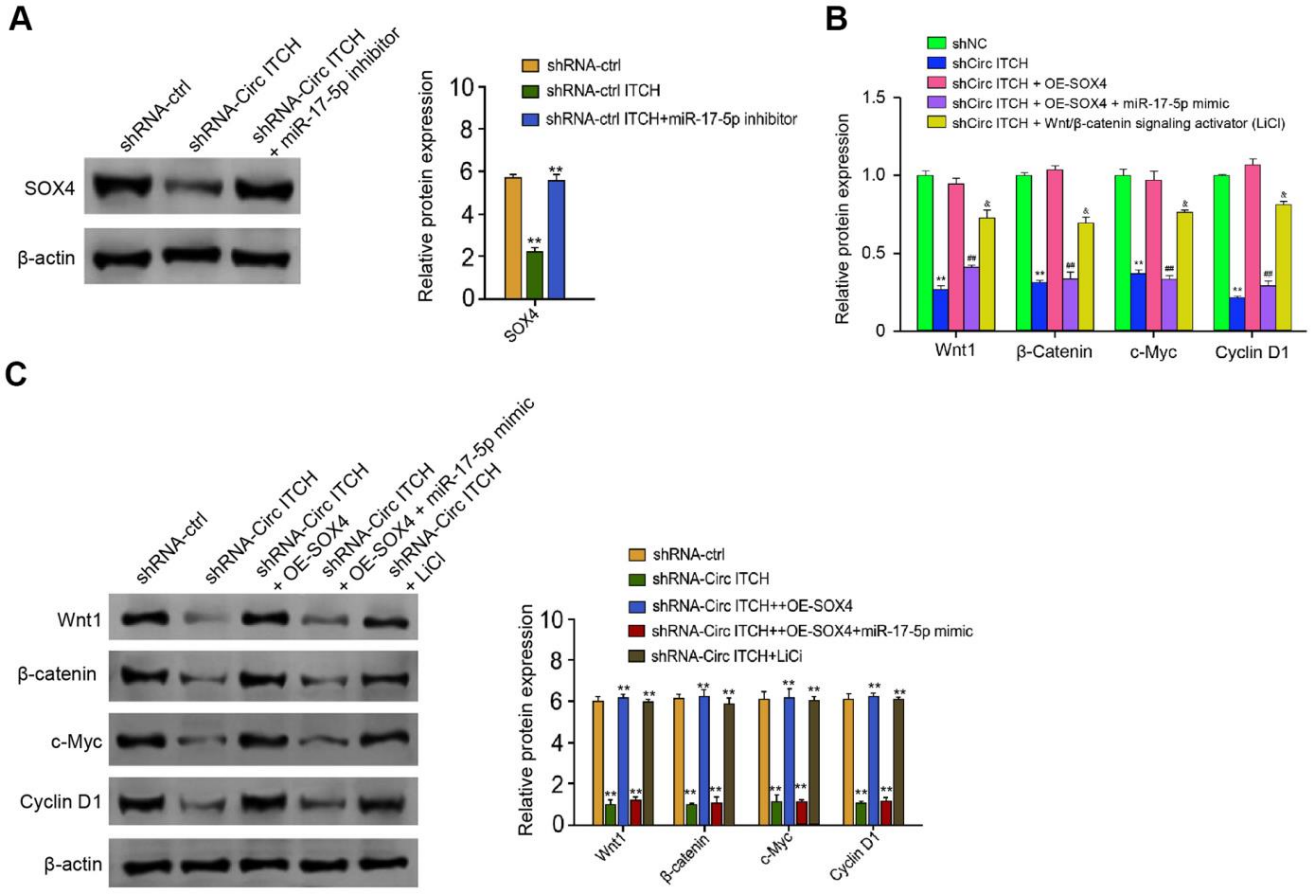
**CircITCH activates Wnt/β-catenin signaling by targeting miR-17-5p/SOX4 axis.** (**A**) The NP cells were transfected with control shRNA, lentiviral plasmids carrying circITCH shRNA, or co-treated with lentiviral plasmids carrying circITCH shRNA and miR-17-5p inhibitor. The protein expression of SOX4 and β-actin was tested by Western blot analysis in the cells. (**B**, **C**) The NP cells were transfected with control shRNA, lentiviral plasmids carrying circITCH shRNA, or co-treated with lentiviral plasmids carrying circITCH shRNA and pcDNA3.1-SOX4 overexpression vector, lentiviral plasmids carrying circITCH shRNA, pcDNA3.1-SOX4 overexpression vector, and miR-17-5p mimic, or lentiviral plasmids carrying circITCH shRNA and LiCl. The expression of Wnt1, β-catenin, c-Myc, Cyclin D1, and β-actin was analyzed by Western blot analysis in the cells. The results of Western blot analysis were quantified by ImageJ software. Data are presented as mean ± SD. Statistic significant differences were indicated: * *P* < 0.05, ** *P* < 0.01.

Next, depletion of circITCH reduced expression of SOX4, in which the miR-17-5p inhibitor reversed the phenotype (Figure 6A). Moreover, we found that the expression of Wnt1, β-catenin, c-Myc, and Cyclin D1 was inhibited by circITCH knockdown, and the overexpression of SOX4 or Wnt1/β-catenin signaling activator LiCl could rescue the expression, in which the co-treatment of miR-17-5p mimic further reversed the effect (Figure 6B, 6C). Taken together, these data suggest that circITCH can activate Wnt/β-catenin pathway by targeting miR-17-5p/SOX4 signaling.

### CircITCH contributes to ECM degradation of degenerative NP cells by miR-17-5p/SOX4/Wnt/β-catenin axis

**Figure 7:**
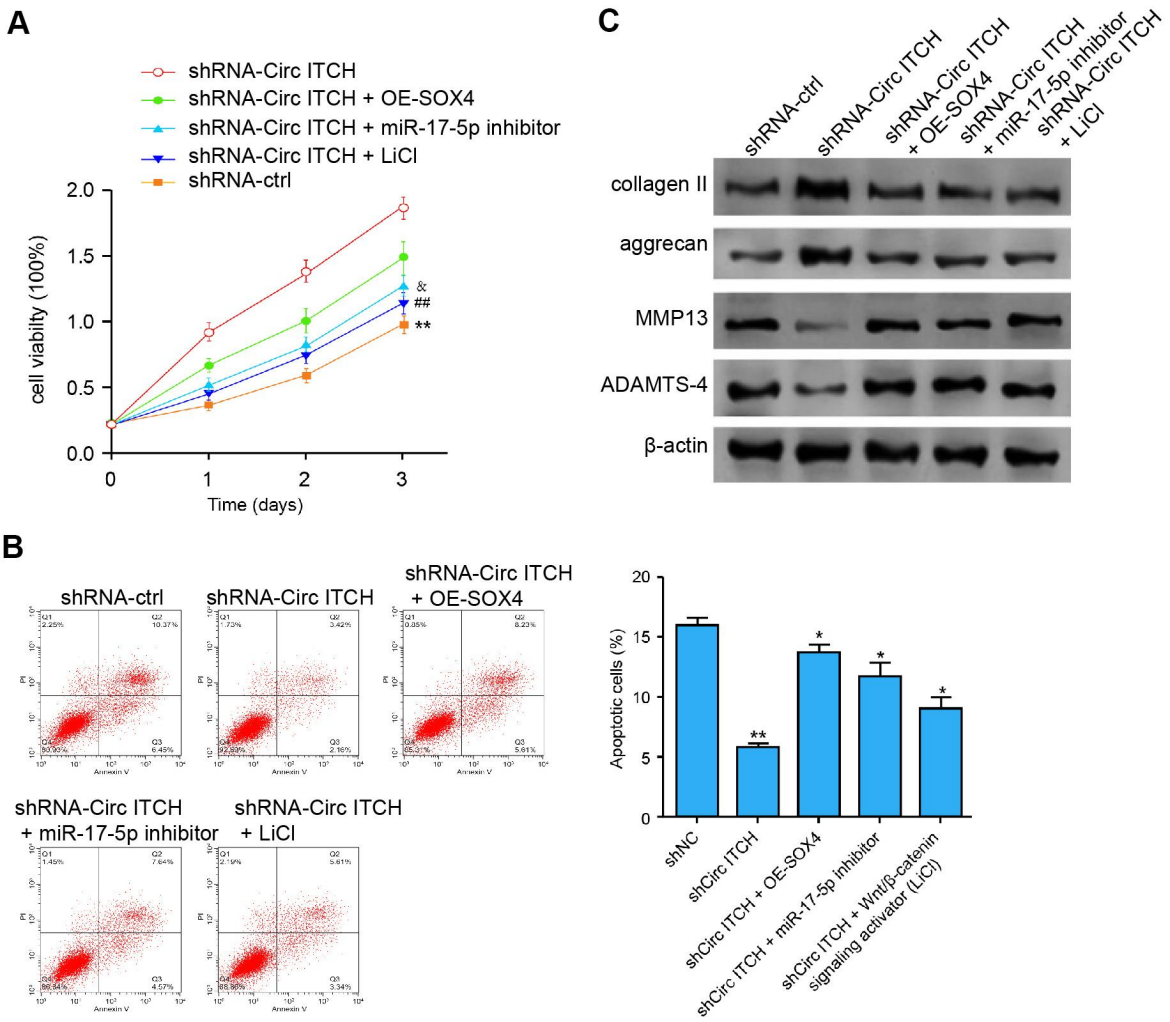
**CircITCH contributes to ECM degradation of degenerative NP cells by modulating miR-17-5p/SOX4/Wnt/β-catenin signaling.** (**A**–**C**) The NP cells were transfected with control shRNA, lentiviral plasmids carrying circITCH shRNA, or co-treated with lentiviral plasmids carrying circITCH shRNA and pcDNA3.1-SOX4 overexpression vector, miR-17-5p inhibitor, or LiCl. (**A**) The cell proliferation was analyzed by CCK-8 assays in the cells. (**B**) Cell apoptosis was tested by flow cytometry analysis in the cells. (**C**) The protein expression of collagen II, aggrecan, MMP13, ADAMTS4, and β-actin was analyzed by Western blot analysis in the cells. Data are presented as mean ± SD. Statistic significant differences were indicated: * *P* < 0.05, ** *P* < 0.01.

We further explored the role of the miR-17-5p/SOX4/Wnt/β-catenin signaling in the circITCH-mediated ECM degradation of NP cells. Significantly, the depletion of circITCH enhanced the NP cell proliferation, in which the SOX4 overexpression, miR-17-5p inhibitor, or LiCl could block the enhancement (Figure 7A). Meanwhile. the SOX4 overexpression, miR-17-5p inhibitor, or LiCl was able to rescue circITCH knockdown-inhibited apoptosis of NP cells (Figure 7B). Moreover, the expression of aggrecan and collagen II was enhanced while the expression of MMP13 and ADAMTS4 was reduced by circITCH depletion, in which the SOX4 overexpression, miR-17-5p inhibitor, or LiCl could reverse the effect (Figure 7C). Together these indicate that circITCH contributes to ECM degradation of degenerative NP cells by miR-17-5p/SOX4/Wnt/β-catenin axis.

## DISCUSSION

IDD serves as the principal contributor to low back pain, in which ECM degeneration is an essential hallmark [26]. CircRNAs are involved in the modulation of IDD. Nevertheless, the role of circITCH in IDD progression and ECM remain obscure. In this study, we identified that circITCH promoted the ECM degradation of IDD by regulating miR-17-5p/SOX4/Wnt/β-catenin signaling.

Multiple circRNAs are involved in the modulation of IDD. Circ4099 is activated by TNF-α and modulates ECM by inhibiting miR-616-5p and up-regulating SOX9 in IDD [27]. CircERCC2 ameliorates IDD by modulating apoptosis and mitophagy via miR-182-5p/SIRT1 signaling [28]. CircSEMA4B controls IL-1β-related degradative alterations in NP cells in IDD by targets miR-431/Wnt signaling [17]. CircVMA21 inhibits IDD via regulating miR-200c [29]. CircTIMP2 serves as a competing endogenous RNA and modulates IDD through mediating matrix metalloproteinase two and miR-185-5p [30]. Here, we identified that circITCH was elevated in the IDD patients. CircITCH enhanced NP cell apoptosis and attenuated NP cell proliferation. CircITCH contributed to the ECM degradation of degenerative NP cells. These data elucidate a novel role of circITCH in the IDD development, providing crucial evidence of the function of circRNAs in the IDD progression. Meanwhile, whether circITCH is the most evident circRNA that was found to be overexpressed and the clinical significance of circITCH in IDD remain unclear and need to be explored.

As a fundamental component of non-coding RNA and the significant interplay factors with circRNAs in various pathological processes, miRNAs are also involved in the modulation of IDD. It has been reported that the silencing of microRNA-221 relieves the IDD in the intervertebral disc cells [31]. MiR-143 increases the progression of NP cell apoptosis by directly targeting BCL2 [32]. Circ104670 presents a crucial role in IDD by serving as the ceRNA of miR-17-3p [33]. MicroRNA-143-5p-targeted eEF2 modulates IDD by the AMPK signaling [34]. It has been reported that SOX4/ Wnt/β-catenin signaling contributes to cancer development [35]. Moreover, Wnt/β-catenin signaling activation and up-regulation of SOX4 contribute to ECM and IDD progression [36–39]. We showed that miR-17-5p attenuates ECM degradation in the NP cells by targeting SOX4. It provides valuable information that miRNAs are involved in the regulation of IDD development. Our mechanical investigation revealed that the Wnt/β-catenin signaling was inhibited by circITCH knockdown, and the overexpression of SOX4 or Wnt1/β-catenin signaling activator LiCl could rescue the expression, in which the co-treatment of miR-17-5p mimic further reversed the effect. The circITCH depletion enhanced the NP cell proliferation and reduced apoptosis, in which the SOX4 overexpression, miR-17-5p inhibitor, or LiCl could reverse the enhancement. The expression of aggrecan and collagen II was enhanced while the expression of MMP13 and ADAMTS4 was reduced by circITCH depletion, in which the SOX4 overexpression, miR-17-5p inhibitor, or LiCl could reverse the effect. These data indicate that SOX4/Wnt/β-catenin pathway was involved in circITCH/miR-17-5p-mediated ECM degradation of degenerative NP cells, in which the circITCH up-regulates SOX4 expression by targeting miR-17-5p. These data uncover an unreported correlation of SOX4/Wnt/β-catenin pathway with circITCH and miR-17-5p and provide new evidence that SOX4 is critical for the modulation of IDD.

In conclusion, we discovered that circITCH promoted ECM degradation in IDD by activating Wnt/β-catenin pathway through miR-17-5p/SOX4 signaling. Our investigation presents novel insights into the mechanism that circITCH regulates the IDD progression. CircITCH, miR-17-5p, and SOX4 may serve as potential targets for IDD therapy.

## MATERIALS AND METHODS

### IDD clinical samples

A total of 90 NP tissues from IDD patients and 90 healthy samples were obtained from the Second Affiliated Hospital of Zhejiang University School of Medicine. All cases were diagnosed by magnetic resonance imaging (MRI) and clinical symptoms. The samples obtained from the patients and normal cases were immediately frozen into the liquid nitrogen, followed by storing at -80° C before further analysis. The samples used in this study were under the written approval by the patients and healthy cases. This study conformed to the experimental guidelines of the World Medical Association and the Ethics Committee of the Second Affiliated Hospital of Zhejiang University School of Medicine.

### Cell culture

The NP tissues were carefully collected from the disc of clinical samples at an aseptic condition, washed by PBS, and separated by surgical scissor. Then, the tissue was digested with collagenase II (0.025%, 4 hours, Invitrogen, USA), followed by filtrating and centrifugation (500 g, 10 minutes). The precipitation was collected. NP cells were incubated at 37° C with 5% CO_2_ in DMEM (GE, USA) containing FBS (15%, Gibco, USA), streptomycin (0.1 mg/mL, Solarbio, China) and penicillin (100 units/mL, Solarbio, China). The lentiviral plasmids carrying circITCH shRNA, the corresponding control shRNA, the pcDNA3.1-circITCH overexpression vector, the lentiviral plasmids carrying SOX4 shRNA, the corresponding control shRNA, the pcDNA3.1-SOX4 overexpression vector, miR-17-5p mimic and inhibitor were obtained (GenePharma, China) (GenScript, China). The Wnt/β-catenin signaling activator LiCl (Aladdin, China) was used at the dose of 10 mM. The transfection in the cells was performed by Liposome 3000 (Invitrogen, USA).

### Quantitative PCR (qPCR)

Total RNA was isolated using TRIZOL Solarbio, China) and the first-strand cDNA was manufactured (TaKaRa, China). The qPCR was carried out via applying SYBR-Green (Takara, China). The primer sequences are as follows: CircITCH 5′-AGGATCCCAGGAGTTCAAAT-3′, 5′-GAGTGGGCTTGACTGAAATAG-3′; miR-17-5p 5′-ACACTCCAGCTGGGCAAAGTGCTTACAGTGC-3′, 5′-TGGTGTCGTGGAGTCG-3′; SOX4 5′-AGGCTCTGAGAAACCTCGGGAAA-3′, 5′-AATGGACATTTACGGTAGTGGGGGAAG -3′; Collagen II 5′-CTGGTGATGATGGTGAAG-3′, 5′-CCTGGATAACCTCTGTGA-3′; Aggrecan 5′-GTGGGACTGAAGTTCTTG-3′, 5′-GTTGTCATGGTCTGAAGTT-3′; MMP13 5′-ACTGAGAGGCTCCGAGAAATG-3′, 5′-GAACCCCGCATCTTGGCTT-3′; ADAMTS4 5′-ACCCAAGCATCCGCAATC-3′, 5′-TGCCCACATCAGCCATAC-3′; GAPDH 5′-TATGATGATATCAAGAGGGTAGT-3′, 5′-TATGATGATATCAAGAGGGTAGT-3′; U6 5′-CTCGCTTCGGCAGCACA-3′, 5′-AACGCTTCACGAATTTGCGT-3′.

### CCK-8 assays

The proliferation was assessed using CCK-8 assays. About 1×10^3^ cells were plated in 96-well dishes and incubated for the transfection or treatment. After 24, 48, 72, 96, and 120 hours, the cells were added with a CCK-8 solution (KeyGEN Biotech, China) and culture for another 2 hours at 37° C. The proliferation was measured at a absorbance of 450nm by applying the ELISA browser (Bio-Tek EL 800, USA).

### Analysis of cell apoptosis

About 2 × 10^5^ cells were plated on 6-well dishes. Cell apoptosis was tested by utilizing the Annexin V-FITC Apoptosis Detection Kit (Keygen, China). Shortly, about 2 × 10^6^ collected and washed cells collected by binding buffer and were dyed at 25° C, followed by flow cytometry analysis.

### Luciferase reporter gene assay

The luciferase reporter gene assays were carried out by using the Dual-luciferase Reporter Assay System (Promega, USA). The cells were transfected with the pmirGLO or pmirGLO-FLG, and LINC01215 siRNA or ZNF652 siRNA by using riboFECT^TM^ CP Transfection Kit (RiboBio, China), followed by the analysis of luciferase activities based on the Dual-luciferase Reporter Assay System (Promega, USA). As control, the luciferase activities of Renilla were measured.

### Western blot analysis

RIPA buffer (CST, USA) was used to extract the total protein, followed by the quantification based on the BCA method (Abbkine, USA). The proteins at same concentration were subjected in SDS-PAGE and transferred (PVDF, Millipore, USA), followed by the incubation with 5% milk and with the primary antibodies at 4° C overnight. The corresponding second antibodies (BOSTER, China) were used for incubating the membranes 1 hour at room temperature, followed by the visualization by using chemiluminescence detection kit (Beyyotime, China). The primary antibodies applied in this study comprising SOX4 (Abcam, USA), collagen II (Abcam, USA), aggrecan (Abcam, USA), MMP13 (Abcam, USA), ADAMTS4 (Abcam, USA), Bax (Abcam, USA), caspase3 (Abcam, USA), cleaved-caspase3 (Abcam, USA), caspase9 (Abcam, USA), cleaved-caspase9 (Abcam, USA), β-actin (Abcam, USA).

### Statistical analysis

Data were presented as mean ± SD, and the statistical assessment was conducted based on GraphPad prism 7. Student’s *t*-test was used to compare two group. *P* < 0.05 were considered as statistically significant.
